# Carbon Footprint Impact, of Monoclonal Antibodies for Severe Asthma, Administered in Italy

**DOI:** 10.3390/biomedicines13071574

**Published:** 2025-06-27

**Authors:** Diego Bagnasco, Laura Pini, Benedetta Bondi, Carola Montagnino, Elisa Testino, Veronica Capuano, Celeste Pugliaro, Luisa Brussino, Stefania Nicola, Marco Caminati, Ilaria Baiardini, Fulvio Braido

**Affiliations:** 1 Respiratory and Allergy Clinic, IRCCS Ospedale Policlinico San Martino, 16132 Genoa, Italy; carola.montagnino@gmail.com (C.M.); testinoelisa@gmail.com (E.T.); cpugliaro@gmail.com (C.P.); ilaria.baiardini@libero.it (I.B.); fulvio.braido@unige.it (F.B.); 2 Department of Internal Medicine (DIMI), University of Genoa, 16132 Genoa, Italy; 3 Respiratory Medicine Unit, ASST-Spedali Civili, 25123 Brescia, Italy; laura.pini@unibs.it; 4 Department of Clinical and Experimental Sciences, University of Brescia, 25123 Brescia, Italy; 5 School of Medicine and Surgery, University of Milano-Bicocca, 20126 Monza, Italy; v.capuano2@campus.unimib.it; 6 SCDU Immunology and Allergology, AO Ordine Mauriziano, 10128 Turin, Italy; luisa.brussino@unito.it (L.B.); stefania.nicola@unito.it (S.N.); 7 Department of Medicine, University of Verona, 37129 Verona, Italy; ma.caminati@gmail.com

**Keywords:** carbon footprint, severe asthma, biologics, CO_2_, environment, monoclonal antibodies

## Abstract

**Background**: Severe asthma is a respiratory condition, involving treatments (i.e., inhaled steroids, systemic steroids, hospitalization) capable of increasing significant carbon footprint, raising concerns about environmental sustainability in healthcare. Sustainable healthcare policies and use of environmentally friendly treatment options are crucial in balancing effective asthma management with climate responsibility. **Objectives**: With this manuscript, we want to assess the impact, in terms of CO_2_ production, of patients suffering from severe asthma and treated with biological drugs, to show the reduction in carbon footprint after the use of these drugs compared to the time when they were not prescribed. We analyzed data from three studies, all conducted in real life in Italy, of patients treated with mepolizumab, benralizumab and dupilumab, for the control of severe asthma. **Methods:** Data on number of exacerbations and hospitalizations, systemic corticosteroids (CS) cycles and their dose, were collected by three already published real-life trials, on the above-mentioned biologics, and used to calculate carbon footprint impact before and after biological therapy. For the mepolizumab study, the data collected referred to patients who started the drug between June 2017 and January 2019; for dupilumab, there were no age limits with patients enrolled between December 2019 and July 2020, whereas in the benralizumab study, all patients had to be over 18 years old. The statistical analysis was performed with Shapiro–Wilk test, *t* test and Cohen’s test. **Results**: The use of biologic drugs showed a significant reduction in CO_2_ production after the introduction of these therapies, mainly secondary to a reduction in exacerbations, hospitalizations and CS use. In numerical terms, an average reduction of 75% in CO_2_ production, per patient, is shown. **Conclusions:** Disease control, clinical remission of disease, in patients with severe asthma is certainly a determining factor in assessing the effectiveness of a treatment. Provided these goals are achieved, biological drug therapy has also proved to be particularly virtuous from the fundamental environmental point of view, allowing a significant reduction in CO_2_ production for the management of these patients.

## 1. Introduction

Asthma is a chronic respiratory disease affecting over 300 million people worldwide, with a growing prevalence in the last decades [1]. The disease imposes a significant health burden, leading to over 400,000 deaths annually, as well as a substantial economic impact on healthcare systems and individuals alike [2]. While the focal point of asthma management is traditionally focused on optimizing patient outcomes, reaching control [2] and remission of the disease [3,4,5], there is a growing recognition of the environmental consequences of the interventions employed in the management and control of the symptoms of this pathology. In last decades, the introduction of biological drugs for the control of asthma has provided to the reduction in oral or systemic corticosteroids (CS), exacerbations and hospitalizations, leading to a better control of the disease [6]. Usually, the efficacy of asthma therapy is evaluated on the above-mentioned goals (exacerbations, CS reduction, improvement of symptoms); sometimes the economic impact of the drugs [7,8], particularly biological, was also evaluated. What is most often misunderstood is the impact of therapies on environment and specifically on the so called “*carbon footprint*”, defined as the total carbon emissions associated with an activity, both directly and indirectly, or the cumulative emissions of a product throughout its life cycle, typically expressed in terms of carbon dioxide (CO_2_) equivalents [9]. Asthma management and its related symptoms also have an environmental impact.

The literature has already expressed on the fact that one of the contributors to the carbon footprint of asthma management is the widespread use of pressurized metered-dose inhalers (pMDIs), primarily due to their propellants [10,11,12]. This focus has largely been on the environmental impact of pMDIs used as maintenance therapy but particularly on the extensive use of short-acting beta-agonists (SABAs) for as-needed symptom relief. Despite GINA guidelines recommending against this approach in favor of SMART (Single inhaler Maintenance And Reliever Therapy), SABAs remain the most widely adopted inhalation therapy in many European countries, even for maintenance. This prevalent SABA-centric therapeutic strategy significantly increases greenhouse gas emissions. Poor symptom control, however, not only results in increased use of SABA, but also in increased use of nebulization therapy and the use of antibiotics and CS therapy, with the subsequent health impact brought about by the repeated and prolonged use of this drug class [13,14,15].

The advent of biologic therapies has demonstrably improved asthma symptom control, leading to a reduction in the use of reliever medications, antibiotics, and CS for symptom management. This has also resulted in a decrease in emergency room visits and hospitalizations for asthma exacerbations. However, limited research has investigated the environmental impact, particularly concerning CO_2_ emissions, associated with the widespread adoption of biologics.

This study intends to direct its attention to a less frequently addressed point: the environmental impact of biological therapies and their ability to reduce carbon dioxide emissions through a reduction in the number of exacerbations. Indeed, the influence of asthma on the environment is not confined to the use of inhalers but also extends to other issues, including the use of add-on medications to baseline treatment, escalation of inhaled therapy, and hospital admissions. Moreover, the total carbon footprint is influenced by the manufacturing, shipping, and disposal of medical equipment, as well as the larger energy requirements of healthcare institutions. For this reason, poorly controlled asthma exacerbations can result in emergency room (ER) visits and hospitalizations, which raises emissions related to healthcare use even more [16].

It is also well known that severe asthmatic patients are the ones with the main impact on the healthcare system, in terms of economic burden. With the possibility to use biologics for the treatment of severe asthmatic patients, we assist in a reduction in CS use and a better control of disease and a decrease of hospitalization as well as exacerbations treated at home. This leads to a decrease in carbon dioxide emissions and a consequent lower impact on the environment. In the field of biologics, we focused more on the environmental impact of those acting on eosinophils, both interacting with interleukin (IL) 5 or its receptor (IL-5r), respectively, with mepolizumab and benralizumab, and the one against receptor of IL-4, dupilumab. This manuscript aims to study how the efficacy of the above-mentioned drugs on exacerbations, hospitalizations and CS sparing effect shows a positive impact on CO_2_ emission reduction in both clinical and real-life studies [17,18,19,20,21,22,23,24,25,26,27,28].

Hence, optimizing asthma treatment not only benefits patients’ health but also has the potential to reduce the environmental burden. The primary endpoint of this study is to assess the impact of biologic drugs from a carbon footprint perspective, analyzing their effect in term of savings of CO_2_ produced for the management of severe asthma. In the patients with severe asthma, succeeding in breaking the vicious circle of poor control-relapse-use of CS, within which hospitalizations can also be included, certainly goes in the direction of virtuosity, from a health, economic and environmental point of view.

## 2. Materials and Methods

### 2.1. Data Analyzed

For this manuscript, we use data about exacerbations, hospitalizations and CS use, shown from three real-life publications, carried out in Italy, of mepolizumab [17], benralizumab [29] and dupilumab [30]. We chose to analyze data of patients from the same country to make the patient cohort homogeneous and make the data on costs and average length of hospitalization more consistent. To ensure more solidity of data, we used two studies from the national severe asthma register (SANI, Severe Asthma Network Italy) [17,30] and a retrospective observational study (ANANKE) [29]. For the mepolizumab study, patients begin the drug from June 2017 to January 2019, and for Dupilumab, between December 2019 and July 2020, whereas in the benralizumab study, it was not specified. The baseline characteristics of the patients being analyzed are summarized in Table 1.

No data about reslizumab were used because it is not marketed in Italy, as well as Tezepelumab because no data about real life at one year, in Italy, are available.

### 2.2. Environmental Impact Data

Regarding data about the environmental impact, we search the literature for similar manuscripts [31,32,33,34], researching what other authors use to make analogue calculations, and then we use the found parameters to “translate” in CO_2_ production the number of hospitalizations, the impact of CS used for exacerbations and the impact of travelling to hospital and hospital visits, from the manuscripts about the three chosen biologics. To calculate the duration of hospitalization, as mean of days of hospitalization for asthma, we use data according to Italian Higher Institute of Health [35].

### 2.3. Statistical Analysis

To check whether the data sample follows a normal distribution, the Shapiro–Wilk normality test was initially performed. Once, the normality distribution of our sample data was confirmed, we used a paired-samples *t* test to compare the data of single variables. Finally, Cohen’s d in this analysis was, in addition, calculated to quantify the power of effect of treatment in reducing carbon footprint. Values with *p* < 0.005 were considered statistically significant. These analyses were performed using statistical program Jamovi® (version 2.3.28).

## 3. Results

Data from the studies cited in the Section 2 show an average production per patient, prior to the use of biologics, of about 181 kg CO_2_/year for the management of asthma-related events, considering the overall emissions of CS use, exacerbations and hospitalizations. The main responsibilities for the CO_2_ production are linked to CS use, both in terms of chronic use for the long-term management of the condition and acute use for managing exacerbations, whether treated at home or in the hospital. After one year of Mabs administration, we assisted with a significant reduction in CO_2_ emissions, from the abovementioned 181 kg/patient/year to 46 kg/patient/year, principally due to the important reduction in hospitalization and CS use, with an average 75% reduction in CO_2_ emissions across all biologics in the studies with a *p* value = 0.032 and Cohen’s d = 2.15. No statistical difference was found between biologics efficacy in emission gain. All additional evidence can be found in Table 2. This means that using biologics drugs allows us to gain 135 kg/patient/year of CO_2_ emissions. This gain, exemplified in everyday life, means a reduction in CO_2_ equal to what is shown in Table 3.

## 4. Discussion

The data extracted from the analyzed studies clearly show that all three biologic drugs—mepolizumab, benralizumab and dupilumab—are effective in reducing environmental impact, as demonstrated by the significant decrease in CO_2_ emissions. Notably, no statistical difference was found between the various drugs studied, showing equal efficacy in reducing CO2 consumption. Our analysis highlights an average reduction in emissions from approximately 181 kg CO_2_ per patient per year to 46 kg CO_2_ per patient per year, with a gain of 135 kg/patient/year. This effect is due to the clinical effect of such therapies in reducing exacerbations, decreasing the use of oral corticosteroids (CS) and improving overall disease control in patients with severe asthma [14,26,27].

It is important to emphasize that the calculation of emissions was carried out by considering several factors: the duration of hospitalizations, the energy consumption during visits and patient transportation, integrating data from national registries and sector-specific studies.

As mentioned in the introduction, a major contributor to the carbon footprint in asthma management has traditionally been attributed to the widespread use of inhaled therapies, particularly pressurized metered-dose inhalers (pMDIs), that use hydrofluorocarbon propellants. However, while previous research has primarily focused on the environmental impact of inhalers, our study highlights that the overall management of severe asthma—including the prevention of exacerbations, reduction in CS use and decrease in hospitalizations—plays a critical role in reducing total CO_2_ emissions [44]. We could not analyze the impact of the drug on patients due to insufficient data in the selected manuscripts. Therefore, we shifted our focus to severe asthmatic patients. We did this for two main reasons: first, they are most heavily implicated in resource utilization and, consequently, CO_2_ production; second, there is a scarcity of data in the literature concerning this specific patient category.

Exploring carbon footprint reduction in the treatment of severe asthma, along with disease control and remission [5,42], is an increasingly important goal in the context of environmental sustainability and public health. There is ample evidence in the literature that poor asthma control, whether for a severe form or an uncontrolled form, is associated with increased CO_2_ emissions, specifically 80% of what is emitted by an asthmatic patient [45]. Achieving disease control and remission certainly meet this objective, reducing the use of both economic and environmental resources otherwise spent on disease management (e.g., hospitalization, visits, medication, ambulance transport, travel, etc.). The production of CO_2_ is associated not only with the above-mentioned factors but also with the use of short acting beta-agonist (SABA), believed to be responsible for more than 60% of all emissions produced by an asthmatic patient and more than 90% of emissions related to uncontrolled asthma [15].

We have seen how biological therapies today represent a promising strategy in this context, and, although they require complex production processes, they can have a lower overall carbon footprint due to fewer disease-related complications [45]. An analysis of the data from the studies reviewed showed that the three factors considered, exacerbations, systemic corticosteroid use and hospitalizations, together heavily affect carbon dioxide production. Looking at what emerges after the introduction of biologic drugs into therapy, with the aim of controlling severe asthma, there is an average reduction of 76 percent in carbon dioxide production to cope with the three factors described above. This reduction demonstrated a robust effect size, with a Cohen’s d of 2.15, indicating a two standard deviation decrease. No statistical significance was found between the difference in gain about the drugs taken in consideration; the little differences are due to the baseline characteristics of patients about the three considered factors.

**Figure 1:**
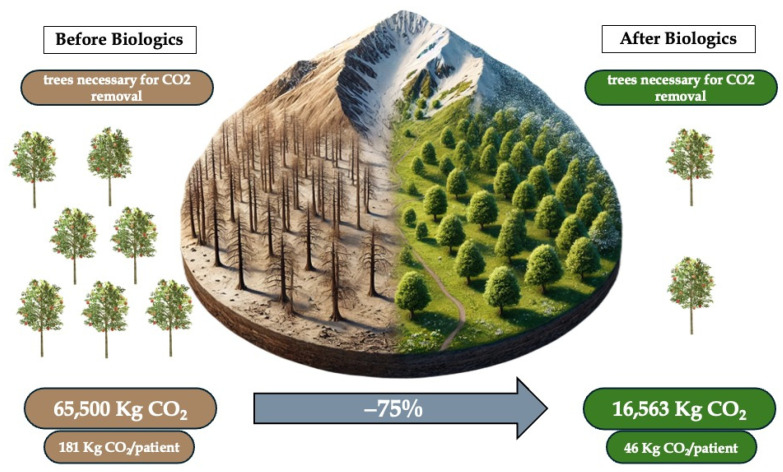
Impact on environment of biological therapy for severe asthma.

To contextualize the findings of this study within botanical principles for enhanced interpretability, we draw upon established data regarding CO_2_ absorption by temperate forest trees. A typical temperate forest tree, such as those found in Italy (where the study data were collected), absorbs approximately 25 kg of CO_2_ per year. Our analysis reveals a mean gain, in terms of equivalent trees, of 599 per year following the introduction of biologic treatments, which translates to an average of 1.7 trees per patient (Figure 1). Given that the biologic drugs considered in this study are known to facilitate long-term disease control, the observed CO_2_ savings are projected to remain consistent over time.

Another noteworthy aspect we want to highlight is the self-administration feature of these drugs. All the biological therapies considered in this study can be self-administered by patients at home. This offers a clear advantage in terms of convenience and ease of managing the therapy, as it can be comfortably taken at home without a scheduled appointment [45]. Secondly, it eliminates the need for patients to travel to the hospital, which not only helps reduce waiting lists but also mitigates the environmental impact of transportation, likely by pollution means, thereby contributing to a reduction in CO_2_ emissions for this purpose. Furthermore, this option confers an additional advantage by reducing travel-related emissions and hospital footfall. In specific healthcare settings, this may equate to hundreds of kilometers of travel avoided per patient per year, with downstream benefits including reduced traffic congestion, lower indirect costs and improved resource allocation.

**Figure 2:**
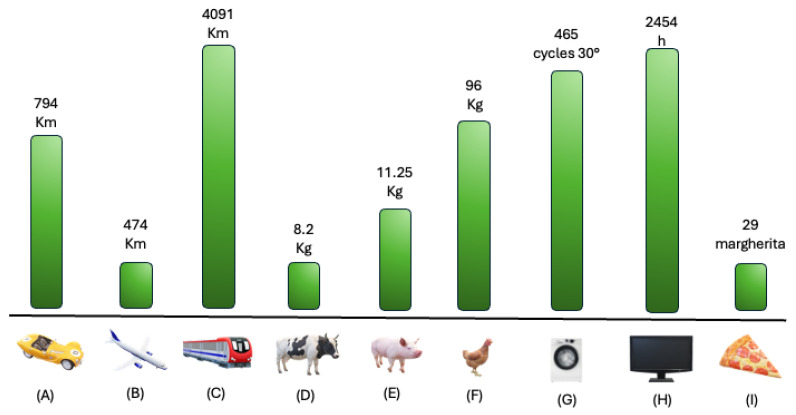
Visualization of yearly gain of CO_2_, in daily routinary samples. Legend. Yearly mean CO_2_ gain, with severe asthma control, converted into common daily life samples. (**A**) Car Km mean between normal fuel, diesel and hybrid/electric car; (**B**) plane; (**C**) train; (**D**) cow meat production; (**E**) pig meat production; (**F**) chicken meat production; (**G**) washing machine cycles at 30°; (**H**) hours of on-demand platforms (i.e., Netflix, Disney+, ecc…); (**I**) number of margherita pizzas.

The implications of these findings are twofold. Clinically, achieving good disease control and even remission is fundamental for improving patient outcomes. Environmentally, reducing the need for emergency care, hospital visits and the associated travel helps to contain healthcare costs and lower the overall emission impact. These results also suggest that future healthcare policies should integrate environmental sustainability objectives into asthma management guidelines, encouraging the adoption of therapies that offer dual clinical and ecological benefits, improving everyday life’s gain of CO_2_ production (Figure 2).

The limitations of our study are primarily related to the scope of our environmental impact analysis of biologics, given the available data. We exclusively evaluated their effect in terms of reduced recourse to CS, hospitalizations and exacerbations. Consequently, we were unable to assess the impact on the reduction in inhaler therapy, the decreased reliance on short-acting beta-agonists (SABAs) or the diminished emissions associated with managing comorbidities linked to prolonged CS use, aspects that would be very interesting to explore. Furthermore, the aspect of home-based administration, which reduces the need for patients to travel to hospital centers and thus impacts CO_2_ emissions from transportation, would be highly insightful to analyze. However, due to the unavailability of such information, this represents an additional limitation to the study, but certainly a very promising and interesting element to investigate further.

In summary, integrating biologic drugs into the management of severe asthma not only leads to significant clinical improvements but also provides considerable environmental benefits by reducing the CO_2_ footprint associated with disease management. This dual benefit underscores the importance of considering ecological outcomes alongside traditional clinical endpoints when evaluating therapeutic strategies for severe asthma. Moreover, further longitudinal studies would be valuable to confirm the environmental sustainability of these interventions on a larger scale, thereby supporting healthcare policies that promote both public health and environmental protection.

## 5. Conclusions

The integration of biologic therapies for severe asthma management represents a significant advancement, offering both substantial therapeutic benefits and an environmentally sustainable approach. Clinically, biologics like mepolizumab, benralizumab and dupilumab have consistently demonstrated a 50–70% reduction in exacerbation rates in real-world settings, coupled with decreased systemic corticosteroid exposure and improved disease control. These improvements enhance patient quality of life, reduce absenteeism and alleviate the socioeconomic burden of uncontrolled asthma. Concurrently, the observed reduction in exacerbations, hospitalizations and oral corticosteroid use directly correlates with a measurable decrease in carbon emissions. This highlights the importance of incorporating environmental metrics into healthcare decision-making and cost-effectiveness analyses. Such an approach supports a shift towards a “green healthcare” paradigm, aligning with both human and planetary health objectives. This also suggests the need for updated clinical guidelines that include environmental sustainability as a therapeutic evaluation criterion. Ultimately, while preliminary observations are promising, further longitudinal, multicenter studies are crucial to validate these findings with comprehensive economic and environmental data. This will guide the adoption of treatment strategies that are not only clinically effective but also environmentally responsible, promoting sustainable, patient-centered care.

## Figures and Tables

**Table 1:** Characteristics of patients at baseline in the three studies.

Characteristics of Patients at Baseline			
Drug	Mepolizumab	Benralizumab	Dupilumab
Sample of patients	157	162	42
Gender (Female, %)	77 (49)	99 (61)	23 (55)
Age mean	59 (21–84)	56 (12.7)	55 (12)
Age Onset	41 (15.7)	-	24 (17)
BMI	25.8 (8.8)	-	26 (4.0)
CRSwNP (%)	99 (63)	86 (53.1)	26 (62)
Exacerbations	3.9 (2.8)	144 (93.5)	2.6 (2.7)
Hospitalizations	1.4 (0.5)	0.2 (0.5)	0.1 (0.3)
CS dependent (%)	85 (54)	41 (25.3)	16 (38)
CS dose g/y	5.8 (4.0)	3.6	2.2 (2.7)
FEV1 %	70 (33)	71 (54–84)	79 (22)
FEV1 L	2.21 (1.0)	1.9 (1.4–2.5)	2.34 (2.7)
FeNO	58 (42)	-	44 (32)
ACT	17 (4)	14 (12–17.5)	17 (5)
SNOT-22	51 (15)	42 (23.0–66.0)	43 (24)

**Table 2:** Production of CO_2_ due to CS use, exacerbations and hospitalization for severe asthma, before and after biological agents use. Number of exacerbations, CS and hospitalization was converted into CO_2_ production according to manuscript data, described in Section 2 [31,32,33,34,35].

**Production of CO_2_e (kg/y) Before Mabs Use**					
Drug	Mepolizumab	Benralizumab	Dupilumab	Total	
Sample of patients (n)	157	162	42	361	
CS (kg/y of CO_2_)	24,613	11,872	3764	40,250	
Exacerbations (kg/y of CO_2_)	323	277	277	877	
Hospitalizations (kg/y of CO_2_)	15,937	2812	5625	24,375	
TOTAL (kg/y of CO_2_)	40,873	14,961	9666	65,502	
CO_2_e production (kg/y)/patient	260	92	230	181	
**Production of CO_2_e (kg/y) post 1 year of Mabs use**				Total	
CS (kg/y of CO_2_)	8977	3764	869	13,610	
Exacerbations (kg/y of CO_2_)	50	33	58	141	
Hospitalizations (kg/y of CO_2_)	1875	0	938	2813	
TOTAL (kg/y of CO_2_)	10,901	3798	1864	16,563	
CO_2_e production (kg/y)/patient	69	23	44	46	
**Reduction in CO_2_ production after 1 year of Mabs use**				Total	*p*-value
Difference pre/post (kg/y of CO_2_)	−29,973	−11,164	−7802	−48,939	0.039 *
Gain of CO_2_e (kg/y) consumption	−73%	−75%	−81%	−76%	0.032 *

* *p*-value is calculated between pre and post all biologics administration, no statistical difference, in CO_2_ gain, was found between the different biologics analyzed.

**Table 3:** Example of gain in CO_2_ production, expressed in everyday life. * On-demand TV (i.e., Netflix, Prime TV, Disney+, DAZN, etc.…).

	Savings with Biologics	Example of Year Gain
Car [36]	794 km	1 Milan-Florence (round trip)
Plain [37]	474 km	1 Milan-Paris
Train [38]	4091 km	1 Madrid-Berlin (round trip)
Cow meat production [39]	8.2 kg	68 Hamburgers
Pig meat production [39]	11.25 kg	562 Slices of ham
Chicken [40]	96 kg	120 Roasted Chickens
Washing Machine [41]	-	465 Washes at 30° 229 Washes at 60°
On-demand platform * [42]	55 g/h	2454 h 1227 days if used 2 h/day 3.3 years if used 2 h/day
Pizza [43]	4.69 kg	29 Margheritas

## Data Availability

The original contributions presented in this study are included in the article. Further inquiries can be directed to the corresponding author(s).

## Abbreviations

The following abbreviations are used in this manuscript:

CO_2_	Carbon Dioxide
IL	Interleukin
CS	Systemic CorticoSteroids
pMDIs	Pressurized metered-dose inhalers
